# Navigating the open innovation paradox: an integrative framework for adopting open innovation in pharmaceutical R&D in developing countries

**DOI:** 10.1007/s10961-022-09958-6

**Published:** 2022-08-18

**Authors:** Bhawani Bhatnagar, Viktor Dörfler, Jillian MacBryde

**Affiliations:** 1 Department of Management Science, University of Strathclyde, Level 7, Sir William Duncan Building, 130 Rottenrow, Glasgow, UK; 2 Innovation and Operations Management, University of Strathclyde, Glasgow, UK

**Keywords:** Open innovation paradox, Patenting, Licensing, Collaboration, M13, L65, L24, O53, O31, O32, O33, O34, O25

## Abstract

In this paper, we combine evidence from eight Indian pharmaceutical firms with extant literature and global best practices to conceptualize an integrative framework addressing the open innovation paradox (OIP), i.e., the tension between intellectual protection and openness. Firms in developing countries face additional challenges in the adoption of open innovation, such as the prevalence of open science norms, weak technology transfer systems, and mistrust between universities and industry; therefore, they employ open innovation selectively for pharmaceutical research. Prior research has examined the strategies to resolve OIP in the context of developed countries; the integrative framework proposed in this paper describes strategies for resolving the OIP in the context of developing countries. This framework illuminates the coping processes of the case firms and provides guidelines to uplift and accelerate the adoption of open innovation strategies in developing countries’ pharmaceutical sectors, and thus provides value to both theory and praxis.

## Introduction

This study contributes to the understanding of the open innovation paradox (OIP), i.e. the tension between intellectual protection and openness in adopting open innovation. Openness causes tensions related to protection of ideas, and puts the potential to profit from innovation at risk (Bogers, 2011; Foege et al., 2019; Lauritzen, 2018; Ritala & Stefan, 2021; Wang et al., 2017). In this paper, we focus on eight Indian pharmaceutical firms in the context of developing countries. We believe that it is important to study open innovation and therefore the OIP within the pharmaceutical industry, as it is a highly regulated area involving high stakes and knowledge intensive processes. Developing countries form an interesting research setting as they play a major role in the global pharmaceutical ecosystem, primarily driven by cost considerations and reverse engineering of drugs. Furthermore, OIP is more significant in developing countries compared to developed countries due to the prevalence of open science norms within academia, poor technology transfer systems, and lack of trust between partners. The reason for choosing the Indian pharmaceutical sector as the study context was that in 2005, India adopted the Trade-Related Aspects of Intellectual Property Rights (TRIPS) agreement, and this regulatory change facilitated a shift in the pharmaceutical sector towards complex new drug research. This provided an interesting research opportunity to observe the behavioral patterns of pharmaceutical firms, and to understand the adoption of open innovation strategies across the phases of pharmaceutical research. Based on a review of the open innovation literature on the global best practices of leading pharmaceutical firms and evidence from case firms, we simplified the main concepts into an integrative framework. The practices proposed in the framework aim to balance the tensions related to OIP and provide guidelines for firms to enact, and in this way uplift the adoption of open innovation strategies in developing countries’ pharmaceutical sectors.

Traditionally, pharmaceutical firms have adopted a closed approach to innovation, leveraging internal research and design capabilities (Peter et al., 2010). As the pharmaceutical industry stands at a crossroads faced with an increase in research costs and decline in R&D productivity, there has been a seismic shift towards adoption of open innovation as an alternate model to enable innovation of new drugs (Chesbrough, 2011; Chesbrough, 2003a, 2003b). Increasingly, firms use inbound open innovation—the practice of exploring and integrating external knowledge from external sources—allowing them to acquire new knowledge. Firms are also using outbound open innovation, which refers to the strategy of establishing relationships with external entities in order to exploit innovation (Bianchi et al., 2011; Chesbrough, 2003a, 2003b; Chiaroni et al., 2009).

Several studies have linked collaboration between public research, large pharmaceutical firms and biotechnology firms to the innovation of novel drugs (Arora & Gambardella, 1990; Cockburn & Henderson, 1996; Owen-Smith et al., 2002). Leading global pharmaceutical firms such as AstraZeneca, GlaxoSmithKline (GSK), Johnson & Johnson, Eli Lilly, Pfizer and Novartis use a variety of open innovation modes such as open source consortiums, crowdsourcing platforms, and virtual R&D to supplement more traditional research collaborations (Gassmann et al., 2008, 2018; Schuhmacher et al., 2016b), however there is little known about the adoption of open innovation in pharmaceutical R&D in developing countries.

At the firm level, appropriability^1^ is a serious concern in the production of scientific knowledge and acts as a double-edged sword (Arora & Gambardella, 1990; Arrow, 1962; Stephan, 1996). The need to protect invention in the pharmaceutical industry—primarily through patenting—has a long tradition (Mansfield, 1986), and it stimulates secrecy among the firms and makes them resistant to open up the process to external actors in the innovation system (Bogers, 2011; Laursen & Salter, 2014). The divergent views on intellectual protection management between academics and corporate R&D restricts academics to forge collaborations with firms, who impose restrictions related to knowledge sharing and exclusivity (Perkmann et al., 2013; West, 2006).

The TRIPS Agreement of 2005, signed by most developing economies like India, South Africa, Brazil and Mexico to bring in intellectual patent protection and unify patent laws, have encouraged pharmaceutical firms to upscale their research to new drug innovation from the traditional manufacturing of generic drugs^2^ (Albuquerque, 1999; Arora et al., 2009a, b; Henry & Lexchin, 2002). Pharmaceutical R&D in developed countries uses basic research from universities to supplement their internal research for innovation (Chesbrough et al., 2006). However, the number of university-industry collaborations in developing countries is low, and they lack experience in technology transfers and management of public–private partnerships (PPP) in contrast to developed countries (Agarwal et al., 2007; Fischer et al., 2018; Mashelkar, 2005; Ye et al., 2013. In case of Indian pharmaceutical sector, academic institutions and firms have worked in silos and research collaborations are relatively few (Agarwal et al., 2007; Lall, 1992). There is also limited evidence of adoption of open innovation in India (Athreye et al., 2009; Narayan & Hungund, 2021; Patra & Krishna, 2015) and a systematic evaluation of the barriers to open innovation is lacking in the literature.

Extant literature on open innovation has focused on the prevalence of the OIP (Arora et al., 2016; Gallagher, 2006; Jarvenpaa & Wernick, 2011; Laursen & Salter, 2014; West, 2006), and suggested mechanisms to cope with the tension (Bogers, 2011; Foege et al., 2019; Lauritzen, 2018) from the perspective of a developed country. There is recent research from developing countries such as China which highlights that extensive reliance on patenting makes firms more dependent on in-house R&D, in turn making it more difficult to form effective collaborative partnerships (Fang, 2011; Wang et al., 2017). The existing literature on Indian pharmaceutical sector has so far focused on R&D intensity, patenting, innovative output (Arora et al., 2009a, b; Chowdhary, 2010; Sampat & Shadlen, 2015), weak technology transfer systems (Agarwal et al., 2007; Lall, 1992) and the effects of open innovation on financial performance (Carayannis & Meissner, 2017; Kafouros & Forsans, 2012; Patra & Krishna, 2015; Shiv, 2016). Therefore, we believe that studying the OIP in pharmaceutical research in developing countries can also yield insights of more general significance.

The current academic literature is lopsided and focuses more on the benefits and adoption of open innovation in pharmaceutical R&D processes (Bianchi et al., 2011; Chiaroni et al., 2009; Filieri et al., 2014; Schuhmacher et al., 2016b, 2018). This paper is motivated by a desire to understand the tensions prevailing in the various phases of pharmaceutical R&D, and to seek ways to accelerate the adoption of open innovation strategies. In this paper, we highlight the specific challenges faced by firms in developing countries practicing open innovation by phase of pharmaceutical R&D, and in this way respond to calls to explore conditions where open innovation fails to work (West and Bogers, 2017; Dahlander & Gann, 2010). This paper fills this gap in the literature by examining the cases of Indian pharmaceutical firms to address the research question: “How do firms in a developing country balance the tension between intellectual protection and openness in the adoption of open innovation in pharmaceutical R&D?”.

Our study provides insights on the “paradox of openness” and makes several contributions to both scholarship and practice. Practitioners and managers in pharmaceutical firms in developing countries can benefit from applying the proposed integrative framework, which allows navigating OIP across the pharmaceutical R&D process. There are numerous countries with a similar context, such as China and Brazil, who have witnessed a shift in patent laws due to TRIPS agreements, and can benefit from the integrative framework proposed in the paper which provides a solid base for strategic consideration. Our findings are important not just for understanding open innovation practices at firm level, but also for policy debates on how to revive the declining public–private interactions between academia and industry. This study brings together different perspectives of academics, public research scientists, and corporate science to provide a holistic view of the common problems inherent in the sector. This provides a solid base to propel policy changes at the highest government level in implementing local scientific collaborations.

This paper has been structured as follows: the first section introduces the background literature underlying this research; the second section introduces the research approach undertaken for the study; the third section presents the empirical findings that describes the open innovation strategies employed by firms to counteract the tensions they face in implementing openness in their R&D process. An integrative framework is presented which can enable pharmaceutical firms to integrate openness while resolving tensions related to OIP. Finally, the implications of these results are discussed, and we draw conclusions from the study.

## Theoretical background

In current times, the focus of innovation has moved a long way from in-house R&D labs to a network of firms. The traditionally closed innovation model that endorses innovation in isolation is fast losing ground, and open innovation in pharmaceutical research is becoming a pivotal innovation strategy (Chesbrough, 2003a, b; Gassmann & Reepmeyer, 2005). An analysis of 798 drug discovery and development projects in the US showed that 32% were collaborative projects, highlighting the importance of universities and biotechnology firms in contributing to the current discovery of innovative drugs (Takebe et al., 2018).

In this section, we first provide a brief overview of the various open innovation strategies across phases of pharmaceutical R&D, and subsequently the OIP, with reference to appropriability. Although, we draw on a broader body of literature, to identify various approaches of open innovation, our focus for analysis is on developing countries—specifically, India.

### Strategies to innovate through open innovation

Pharmaceutical research for new drugs has well-defined steps and can be categorised into two important phases: drug discovery, and drug development. When a drug candidate is formulated after a rigorous exercise of screening and validation, a set of potential therapeutic applications (i.e., compound/targeted disease combinations) are identified in the initial drug discovery phase. The latter phase comprises of drug development that aims to establish the safety and efficacy of the drug through experiments on animal subjects (“pre-clinical trials”) and clinical trials on human subjects (Arora et al., 2009a, b).

**Fig. 1:**
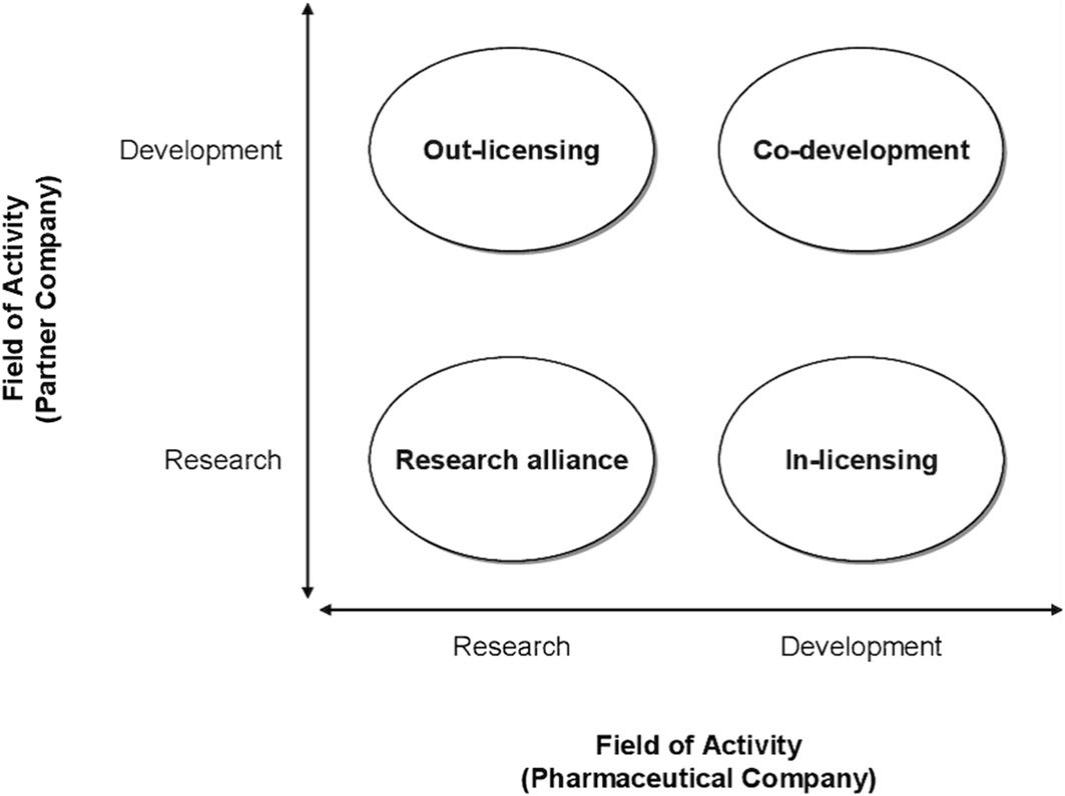
Strategies employed by firms for pharmaceutical innovation. Source: (Reepmeyer, 2006)

In the pharmaceutical industry setting, four types of arrangements are prevalent (see Fig. 1): (a) Research alliance, (b) In-licensing, (c) Out-licensing, and (d) Co-development (Reepmeyer, 2006). Literature on pharmaceutical open innovation shows that inbound innovation through research alliance and in-licensing mostly takes place in the drug discovery phase, i.e. target identification, lead validation and pre‐clinical tests, to leverage highly-specialized competencies, or for in-sourcing an innovation. Outbound innovation takes place through out-licensing and co-development agreements, and is mostly confined to drug development phases i.e. in the clinical tests and post‐approval activities for commercial exploitation of the innovation (Bianchi et al., 2011; Chiaroni et al., 2009).

Research alliance are formed when partners engage in basic research and drug discovery to leverage the expertise of the scientists and steer through the murky drug discovery phase (Reepmeyer, 2006). Research partnerships and research services form important mechanisms to source knowledge from external sources, promote organisational learning, exploit resources, and develop competitive advantage (Hagedoorn et al., 2000; Perkmann & Walsh, 2007). Research services are contracts between two partners focussing on research projects with a defined scope, to avoid unintended spillovers and leverage personal networks (Hagedoorn et al., 2000; Rouyre & Fernandez, 2019).

Crowdsourcing platforms are another innovative way to seek expert opinion and form research alliances, gather and co-create innovative ideas, connect internal R&D scientists to a virtual global community of highly qualified scientists, and provide opportunities to source in new ideas (Schuhmacher et al., 2016a). These platforms are based on the concept of transparency, and provide intellectual property rights to the proposer while allowing them to get solutions to complex drug discovery problems through selective revealing (Cappa et al., 2019; Foege et al., 2019; Schuhmacher et al., 2016a). Crowdsourcing allows firms to engage with the relevant experts in the field in order to harness the collective competence of scientists. Prominent examples of crowdsourcing initiatives include Eli Lily’s Innocentive, Bayer Healthcare’s Grants4Targets and Open innovation platform by Astrazeneca, The Synaptic Leap's Schistosomiasis’ (TSLS) and Open Source Drug Discovery (OSDD) initiated by the Council of Scientific and Industrial Research (CSIR), India (Årdal & Røttingen, 2012; Bhardwaj et al., 2011; Dahlander et al., 2021; Schuhmacher et al., 2016a).

Research partnerships are formal collaborative research activities that typically last longer than a single project and may extend into integrated long-term research multi-projects with multiyear agreements for drug discovery (Perkmann & Walsh, 2007). Largely, research alliances allow firms to retain control of intellectual property and stipulate confidentiality agreements (Perkmann & West, 2014). Large US based pharmaceutical firms such as Merck, GlaxoSmithKline, AstraZeneca and Pfizer have all established multiyear collaborations with academic institutes for a number of therapeutic areas (Melese et al., 2009). Remicade (Infliximab), currently marketed by Johnson & Johnson, was originally discovered by scientists at the New York University (Johnson, 2017). Eli Lily has strategically established a vast network of collaborators and integrated open innovation practices in all steps of drug research to ensure a steady pipeline of new drug compounds. Through the Phenotypic Drug Discovery (PD2) and Target based screening (TargetD2) initiatives, the firm provides access to its essays to academic scientists for screening and lead optimization of their molecules while allowing the innovator to retain intellectual property rights (Hunter, 2014).

Another form of open innovation model is ‘Public–Private Partnership’; essentially a collaboration between industry and academia with funding by the government or a third party. Such PPP programmes aim to initiate research in areas which are not commercially profitable for the private industry to take up, but have high social benefits (Stiglitz & Wallsten, 1999). In the past decade, these partnerships have taken the form of open source philosophy, particularly for neglected diseases, where many organizations pool resources to conduct collaborative research and speed up development of a drug with no legal rights for the contributors (Gassmann et al., 2018). Noteworthy examples include Medicines for Malaria Venture (MMV), The Global Alliance for Tuberculosis Drug Development (TB Alliance), and Drugs for Neglected Diseases (DNDi) (Munos, 2010; Schuhmacher et al., 2018). Some of the large Indian pharmaceutical firms such as Lupin use this route to foster drug innovation for tropical and neglected diseases (Bhardwaj et al., 2011).

Another strategy used to boost R&D for neglected diseases are the use of patent pools in which a group of patent holders license their respective patents to each other and to third parties. Notable example include The Medicines Patent Pool, established in 2010 by the WHO, dedicated to improving the treatment of HIV/AIDS, tuberculosis, and malaria, as well as the US National Institutes of Health (NIH). Gilead and other pharmaceutical firms have also granted free-licenses to the members of the consortium (Ziegler et al., 2014).

In-licensing is a strategy to source knowledge, new drug molecules, or distinctive knowledge service packages, and allows formal transfer of IP rights from the innovator to the firm (Reepmeyer, 2006). In the field of pharmaceutical research, examples include in-licensing of anti-malarial technology by Sanofi Aventis from Amyris, in-licensing of drugs such as Pregabalin (Lyrica), and conjugated estrogens (Premarin) by Pfizer from universities (Chesbrough, 2011; Jung, 2019; Pfizer, 2013; Takebe et al., 2018). Recent releases of Novartis, Aimovig (Erenumab), Ajovy (Fremanezumab), and Emgality (Galcanezumab) were in-licensed respectively from Amgen, Pfizer, and Eli Lilly (Lloyd, 2019). The decline in success rates of the drug discovery projects has encouraged firms to explore the option of acquiring drug discovery targets from academia and public sector. However, in-licensing brings in additional challenges regarding integrating new knowledge with existing knowledge, coping with the ‘not-invented-here^3^’ syndrome, and potential conflicts of ownership of ideas (Chesbrough, 2003a; Chiaroni et al., 2009; Hannen et al., 2019; Lauritzen, 2018).

Out-licensing involves the commercialisation of innovation by firms for pecuniary or monetary benefits (Dahlander & Gann, 2010). Out-licensing strategy also enables a firm to manage its portfolio of drug compounds under limited resources and time constraints. It is an especially useful strategy if the market potential of pipeline compounds is less than the set threshold level (Danzon et al., 2005). An analysis of pharma licensing deals of leading pharmaceutical firms show that Bristol-Myers Squibb and Pfizer have out-licensed approximately 20% of their portfolio of drug compounds (O'Connell et al., 2014).

A co-development agreement is usually formed during the drug development phases and allows leveraging the development and marketing capabilities of the other organisation. Such agreements are usually characterized by revenue sharing or profit sharing agreements (Reepmeyer, 2006). Furthermore, horizontal linkages with other competing firms may help to overcome appropriability problems, as the firms would have collectively contributed to the R&D costs (Teece, 1992). Eli Lily has also established a taskforce unit, Chorus, to push potential drug candidates into drug development using a virtual network of scientists from within and outside the company. The productivity of Chorus has been 3–10 times higher than the traditional pharmaceutical R&D model of Eli Lilly (Gassmann et al., 2008). Despite the recent increase in outbound innovation by firms, it involves risk as it may enable competitors to gain knowledge, may result in loss of market exclusivity, and acquire control over product and patent rights (Chesbrough, 2003a; Kline, 2003; Lichtenthaler, 2008).

The pharmaceutical industry is experimenting with new ways of achieving R&D efficiency and greater return on investment through partnerships, outsourcing, innovation networks and academic alliances (Kaitin & DiMasi, 2010). The boundary of pharmaceutical industry is becoming more permeable as global pharmaceutical firms are experimenting with new models to aid their drug discovery efforts (Bianchi et al., 2011; Chiaroni et al., 2009; Gassmann & Reepmeyer, 2005; Hunter & Stephens, 2010; Olk & West, 2020; Schuhmacher et al., 2013, 2016b). In the case of developing countries, this concept is still in its infancy. There are few empirical studies that suggest that pharmaceutical firms in developing countries are making the transition from insular R&D model to a collaborative model for new drug innovation post-2005 (Athreye et al., 2009; Fischer et al., 2019; Shiv, 2016; Wang et al., 2017). Though firms in developing countries tend to rely on in-house R&D for innovation, they are now opening their boundaries for out-licensing agreements, which has also increased their motivation to patent (Athreye et al., 2009; Narayan & Hungund, 2021; Srivastava & Wang, 2015). The current literature is largely unbalanced however, and mostly focuses on the adoption of open innovation in developed countries.

Basic research in developing countries mostly occurs inside the walls of pharmaceutical laboratories, due to issues of confidentiality, knowledge control, publication, and intellectual property (IP) rights (Wang et al., 2017). Studies on the topic of university-industry relations in developing countries show that there is an environment of mistrust, and therefore research engagements are moderately low (Agarwal et al., 2007; Fischer et al., 2019; Srinivas, 2004; Wang et al., 2017; Ye et al., 2013). There is emerging evidence in the literature about the participation of pharmaceutical firms of developing countries in research consortia primarily for neglected diseases such as Open Source Drug Discovery (OSDD) (Schuhmacher et al., 2018) and Medicines for Malaria venture (Nwaka & Ridley, 2003). Although, this is a positive step for open innovation, the frequency of such collaborations is lower, as neglected diseases do not form the core business of pharmaceutical firms in both developing and developed countries (Schuhmacher et al., 2018). Table 1 lists the types of open innovation strategies used in developed and developing countries based on review of literature.

**Table 1:** Types of open innovation strategies used in developed and developing countries

Phase of pharmaceutical research	Open innovation strategies	Description	Open innovation in developed countries	Open Innovation in developing countries
Drug discovery	Research alliance	Research services – Contracts between two partners focusing on research projects with a defined scope (Hagedoorn et al., 2000; Rouyre & Fernandez, 2019)	Universities form an important source for knowledge sourcing (Owen-Smith et al., 2002; Perkmann & West, 2014; Powell, 1998; Theeranattapong et al., 2021)	In-house R&D predominant in pharmaceutical sector to retain exclusive ownership of IP (Athreye et al., 2009; Narayan & Hungund, 2021; Srivastava & Wang, 2015)
		Crowdsourcing platforms- seek expert opinion from virtual global community of scientists (Schuhmacher et al., 2016a)	Crowdsourcing used to engage with scientists for drug discovery problems by global pharmaceutical firms (Dahlander et al., 2021; Schuhmacher et al., 2016a)	CSIR initiated Open Source Drug Discovery to enable scientific community to collaborate virtually through open source projects (Årdal & Røttingen, 2012; Bhardwaj et al., 2011)
		Research Partnerships—long-term research projects with multiyear agreements for drug discovery (Perkmann & Walsh, 2007)	Large pharmaceutical firms have established multiyear collaborations with academic institutes (Hunter, 2014; Johnson, 2017; Melese et al., 2009)	Environment of mistrust and research engagements are moderately low (Agarwal et al., 2007; Fischer et al., 2019; Srinivas, 2004; Wang et al., 2017; Ye et al., 2013) Lack of inter-firm co-operation between different agents of the innovation system in India (Joseph & Abraham, 2009; Ramani, 2002; Srinivas, 2004)
		Public–Private Partnerships—collaboration between industry and academia with funding by the government or third party (Stiglitz & Wallsten, 1999)	Firms participate in PPP research for not profitable diseases (Munos, 2010; Schuhmacher et al., 2018)	Firms are now participating in PPP for neglected diseases (Schuhmacher et al., 2018)
	In-licensing	Strategy to source in knowledge, new drug molecules or distinctive knowledge service packages (Reepmeyer, 2006)	Universities an important source for in-licensing (Chesrough, 2011; Jung, 2019; Pfizer, 2013; Takebe et al., 2018)	In-licensing agreements are limited (Fang, 2011; Wang et al., 2017)
Drug development	Out-licensing	Commercialisation of innovation by firms for pecuniary or monetary benefits (Dahlander & Gann, 2010)	Used to manage drug portfolio and for commercialisation of innovation by firms (Dahlander & Gann, 2010; O'Connell et al., 2014)	Out-licensing agreements are on the rise to profit from intellectual property (Athreye et al., 2009; Srivastava & Wang, 2015)
	Co-development	Revenue sharing or profit sharing agreement with a partner organisation (Reepmeyer, 2006)	Used by firms to share knowledge, costs and risk (Reepmeyer, 2006; Schuhmacher et al., 2016b)	Firms collaborate with partners to commercialise innovation (Fischer et al., 2019; Arora et al., 2009a, 2009b;Wang et al., 2017; Shiv, 2016; Athreye et al., 2009)

### Appropriability and open innovation paradox

At the firm level, appropriability is a serious concern in the production of scientific knowledge (Arora & Gambardella, 1990) and appropriability conditions determine the level of profits a firm can make through its innovative activity (Cohen & Levinthal, 1990; Teece, 1986). Patents, copyrights, trademarks, trade secrets, restricted access, contracts, passwords, secrecy are all different forms of appropriability measures which enable an inventor to protect an invention (Hurmelinna & Puumalainen, 2005). A key theoretical perspective highlighted in research is the role of appropriability in influencing open innovation patterns; specifically in the R&D intensive pharmaceutical industry, which has a high rate of innovation, and relies on patenting to protect its investment (Mansfield, 1986). Patenting, a measure of innovation output, supports exploitation of innovation in three ways: (a) it enables protection of invention and can also be used to block rivals from patenting related inventions, also referred to as ‘patent blocking’ (Cohen et al., 2000), (b) it safeguards intellectual property against imitation and facilitates licensing deals, which require information disclosure between buyers and sellers in a secure environment (Gallini, 2002; Granstrand & Holgersson, 2017; West, 2006), and (c) it provides an opportunity for firms to commercialize their inventions in new territories (Archibugi & Michie, 1995).

There are conflicting views on open innovation and patenting in the literature. There are papers which argue that firms who adopt open innovation strategies use patenting as a key strategy to secure protection, freedom to operate and profit from innovation (Arora et al., 2016; Chesbrough, 2003a, 2003b; Granstrand & Holgersson, 2017). In their empirical study, Laursen and Salter (2005) have shown that pharmaceutical industry with high levels of appropriability mechanisms also shows high degree of openness to external knowledge sources. In this regard, tough intellectual property laws enable firms to undertake open innovation without worrying about acts of opportunism and imitation, and hence facilitate profit from innovation. Firms utilizing open innovation strategies undertake extensive patenting to engage in more collaborative agreements (Granstrand & Holgersson, 2017). Research also shows that firms who routinely engage in-licensing activities develop superior licensing and knowledge assimilation capabilities, and this in turn fosters more patenting (Srivastava & Wang, 2015).

A contrasting view also exists which states that strong appropriability measures bring an increased patenting, secrecy and restricts follow-on research (Gallini, 2002). The need to protect knowledge and intellectual property rights (Bogers, 2011) encourages secrecy among firms, and influences boundary tensions between organizations (Ritala & Stefan, 2021). While a strong appropriability strategy solves the problem of protection and provides a secure environment for firms to open their boundaries for innovation, too much reliance on appropriability also limits flow of innovation (West, 2006). It has been well demonstrated in the literature that high levels of appropriability incentivizes firms to restrict interactions with external sources to protect knowledge, thereby missing opportunities to engage and accelerate innovation with different entities in the innovation system (Arora et al., 2016; Laursen & Salter, 2005, 2014).

Universities form an important external source for knowledge sourcing, and drive innovation processes for science-intensive industries such as pharmaceuticals (Owen-Smith et al., 2002; Perkmann & West, 2014; Powell, 1998; Theeranattapong et al., 2021). Firms form partnerships with universities and external partners to access necessary basic research for innovation and knowledge co-creation (Arora & Gambardella, 1990; Theeranattapong et al., 2021). Various path-breaking discoveries in genetic engineering, such as the recombinant DNA method, cell infusion technology, and gene sequencing, have been done in universities, and the spillovers have enabled the pharmaceutical industry to benefit from these research outputs (Powell, 1998). The open science system is the practice that encourages academic researchers to openly publish and distribute their research findings in the hope that interested parties will value and build on their work (Fabrizio, 2006). For years, the goal of scientists has been to communicate their findings in a timely manner and establish priority in the scientific community mainly through publications (Merton & Merton, 1968; Stephan, 1996).

In developed countries, the university systems have seen a transition towards a hybrid system where the principles of industry have been integrated with the tenets of open science (Murray, 2010; Owen-Smith, 2003; Shibayama et al., 2012). University researchers are shifting to proprietary science through patenting, and to the commercialization of research output through technology licensing offices, incubator parks for start-ups, and spin-offs from university laboratories (Siegel et al., 2003; Walsh & Huang, 2014). The Bayh-Dole Act in the US, and similar frameworks in other developed countries have propelled increased patenting of university research, and encouraged licensing to industry, thus earning revenues and reducing dependency on public funds for research (Nezu, 2005; Shibayama et al., 2012). Patenting and industry funding works against the tenets of open science as it is associated with secrecy and publication delay to protect the value of research findings (Walsh & Huang, 2014; West, 2006).

While universities in developed countries are shifting towards proprietary science, open science norms still prevail in developing countries including India, where the university system mainly rewards research publications (Fischer et al., 2018; Walsh & Huang, 2014; Ye et al., 2013). Though university-industry linkages have increased in the 2000’s, there is a lack of high quality R&D-oriented partnerships, and unlike developed economies, collaborations with universities do not play a key role in driving innovation (Fischer et al., 2018). This, coupled with poor infrastructure for technology transfer mechanisms, bureaucracy of the state machinery, and low mobility of scientists between university and industry (Agarwal et al., 2007; Lall, 1992) has resulted in weak interactions between industry, government and universities (Ye et al., 2013). Specifically, in India there is a history of lack of inter-firm co-operation between different agents of the innovation system (Joseph & Abraham, 2009; Ramani, 2002; Srinivas, 2004). The implementation of TRIPS in 2005 has led to mandatory patenting of pharmaceutical products in all developing countries (Sampat & Shadlen, 2015). The change in patent laws encouraged the shift in research activities of Indian firms from R&D of generic drugs to advanced innovative R&D for new drug research (Chaudhuri, 2007; Kale & Little, 2007). It also brought with it changes such as increased budgetary allocations for research, IP awareness programs, and increased thrust by the government to initiate policies to link science with industry and encourage collaborative innovation (Department of Science and Technology, 2013). Despite these initiatives, industry-academia linkages are weak and institutions largely function in silos (Patra & Krishna, 2015). Firms are unwilling to have multiple partners contribute to the innovation in order to retain exclusive ownership of intellectual property (Jarvenpaa & Wernick, 2011). In collaborative research, restrictions are also imposed on academics with respect to knowledge sharing in order to maintain exclusivity (West, 2006). Thus, there exists a situation that is conflicting and not conducive for open innovation.

**Fig. 2:**
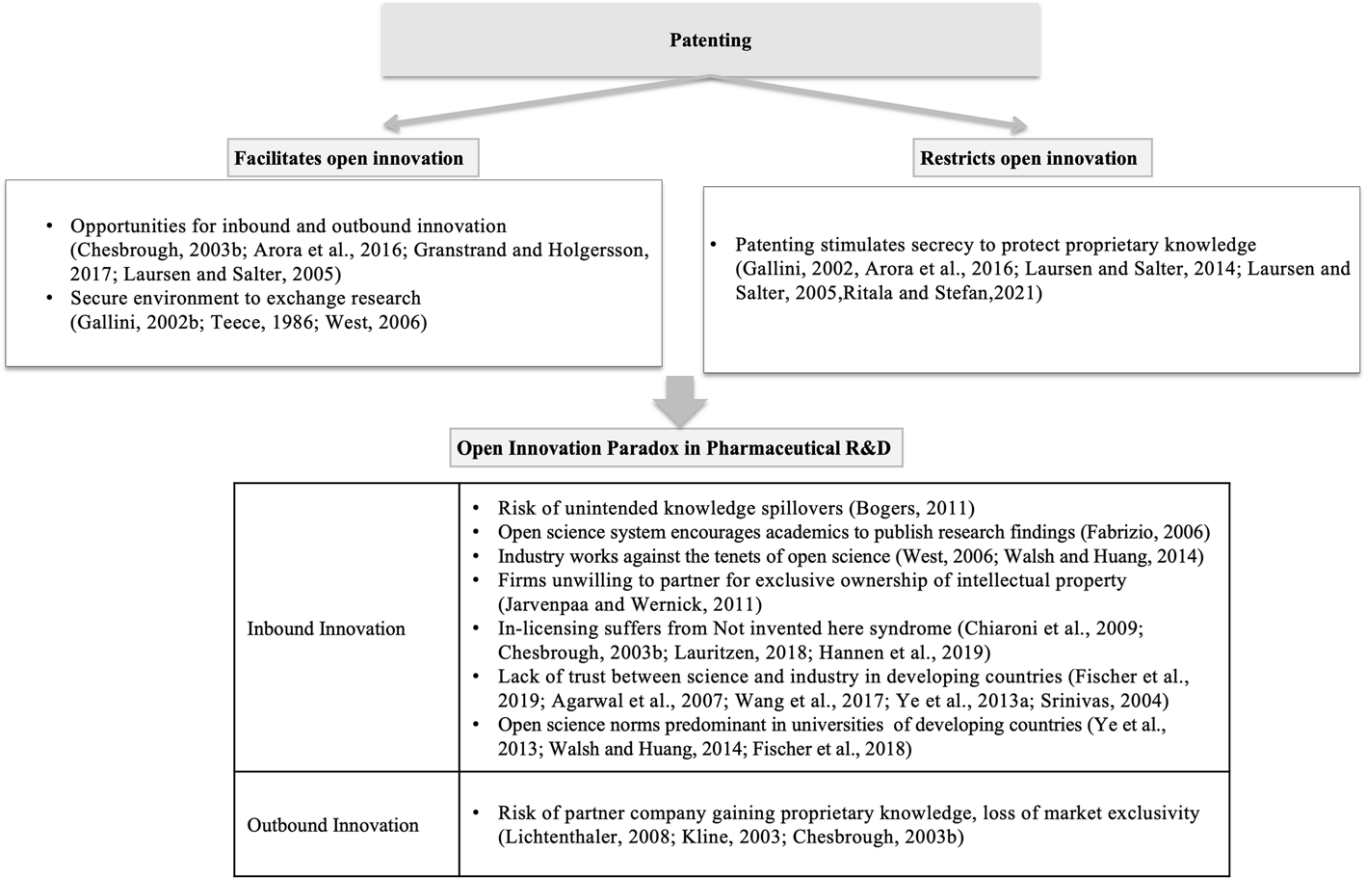
OIP in Pharmaceutical R&D

In essence, industrial sectors employ different strategies to manage the tensions in open innovation collaborations. Firms employ various strategies to navigate the tension field such as pooled R&D, spinouts, restricting open innovation for complementary products (Gallagher, 2006) or by combining differentiation and integration strategies (Lauritzen, 2018). Other strategies include open knowledge exchange through layered collaboration (Bogers, 2011) and configuration of specific appropriation practices based on firm size, trust, and type of collaborative project (Foege et al., 2019; Tekic & Willoughby, 2019). Global leading pharmaceutical firms have also implemented various risk sharing strategies that allow to circumvent the intellectual protection challenges (Gassmann et al., 2018). The scholarly literature still does not offer an adequate understanding of the OIP in pharmaceutical R&D in a developing country setting. We organize the key concepts from the OIP literature to depict the tensions in pharmaceutical R&D in Fig. 2.

## Methods

The literature review in the previous section provided insights on the practices of open innovation by firms in pharmaceutical research, and highlights the conflicting dimensions of openness and patenting mainly from the perspective of developed countries (Refer Table 1 and Fig. 2). In order to address the research question as to how firms in developing country balance the tension between intellectual protection and openness, we studied the Indian pharmaceutical sector between 1995 and 2020 to cover the period before and after TRIPS implementation. This section provides an overview of the research setting, the data collection methods, and the methods used for the analysis.

### Research setting

India implemented the TRIPS-based patent laws on January 1, 2005 (Sampat & Shadlen, 2015), which marked a significant transition point for the pharmaceutical industry, which was heavily dependent on generic business. The shift towards innovative new drug research in a strong patent regime provided an interesting research setting to explore different facets of open innovation in a sector backed by vast scientific infrastructure, making it suitable for a large-scale robust study. The pharmaceutical industry dealing with contemporary issues entails posing ‘what’, ‘how’ or ‘why’ questions that provide a rationale to pursue case study research (Gummesson, 2007). The research design is a qualitative multiple case study, and the units of analysis are the case firms. The time horizon is longitudinal as the change in behavioral patterns of case firms was examined over 25 years. The selected cases vary in type, size, and range of R&D activities and the analysis of the case firms enabled us to understand in depth, their unique contexts and experiences with respect to open strategies and the innovation networks.

The Indian pharmaceutical sector consists of hundreds of large firms and more than 10,000 small to medium drug manufacturers, including start-ups (IBEF, 2022). In order to ensure comparability, we narrowed down the scope to firms that engage in new drug research and development (R&D) of small molecules. At the time of our study, only 15 large generic pharmaceutical firms were involved in such research. There is no published data on the number of start-ups, but extensive secondary research revealed an estimate of 10 start-up Indian firms involved in the research of small molecules (Differding, 2017). Thus, the population of our study includes 15 large firms and 10 start-ups; of these, 5 large firms and 3 start-ups were chosen as the study sample. The sampling strategy was purposive and cases were selected on the basis of who would be eligible and willing to provide useful data (Bryman, 2011). The professional experience of the lead author in the Indian pharmaceutical sector made the access possible and enabled a better correspondence with the respondents by virtue of shared knowledge background (Stierand & Dörfler, 2014).

### Data collection

Our empirical study was based on multiples sources of data (see Table 6): (1) patent applications data (2) qualitative data from semi-structured interviews (3) archival data from annual reports, firm websites, online magazines (Pharmabiz, Express Pharma), government sponsored initiatives^4^ and (4) data from available literature on open innovation strategies of top 20 global pharmaceutical firms^5^ identified by market capitalization. Apart from the interviews, all the data sources are in the public domain.

Semi-structured interviews were carried out with 50 key informants (Chief Scientific Officers, R&D heads of pharmaceutical firms), as well as academics, scientists in public research labs, and public officials leading government sponsored drug research initiatives, to get a comprehensive set of views from the sector. The research participants are listed by position and organization in the Appendix.

The qualitative research process in this study was iterative, and the interviews were conducted over a period of two years, starting in 2013. The semi-structured interviews at the beginning had a wider scope to understand the phenomenon of open innovation, extent and nature of collaboration for new drug research. Such an approach is referred to as “emergent case studies” in contrast with the traditional ones (Lee & Saunders, 2017; Saunders, 2019). The interviews lasted an average of 30–45 min and gradually the interviews became more structured as themes emerged from the data. This progressive focus of the interviews allowed for targeted data collection that enabled the identification of patterns, categories and themes. When no new themes emerged from the data analysis which was done in parallel with the data collection, and when the interviewer, felt that a saturation has been reached, no further interviews were conducted. While there is no rigorous way to determine what the right number of interviews is for a good quality research, the generic guideline is to understand the phenomenon under scrutiny (Pratt, 2009), which we achieved by making use of the lead author’s insiderness (Stierand & Dörfler, 2014). The use of interviews provided a rich opportunity to gain in-depth understanding and allowed to explore large number of issues related to open innovation (Reed & Payton, 1997).

A semi-structured interview guide was developed prior to the interviews; the interviews were organised around a small number of themes (Saunders, 2019). The earlier interviews allowed to adjust the themes for the later interviews. In line with the semi-structured nature of interviews, a great deal of flexibility was given to the participants to talk about their points of interest – what the participants find important also constitutes data. After each interview, the voice recordings were transcribed verbatim, and the data analysis commenced in parallel with the data collection.

### Data analysis

The data analysis was conducted in two main phases, which are presented separately below. However, both these phases were intertwined, mutually informed each other, and multiple iterations occurred.

Phase 1- Reconstructing a chronology of events and building case summary. We combined annual reports of the case firms with archival data to compile a list of collaborative agreements separated by stages of drug research. We used the types of open innovation strategies provided in Table 1 to categorize these collaborations. A similar approach was undertaken for the top 20 global pharmaceutical firms to understand the types of open innovation strategies in developed countries, as a reference point.

Patents are important indicators for innovation as organizations place emphasis on patenting their innovations, especially in the pharmaceutical industry (Mansfield, 1986). The types of patents filed are reflective of the innovative output of a firm. The patent dataset comprising of 780 patent applications of the case firms between 2005 and 2020 were analyzed to estimate the number of product patents filed by the firms. Table 2 provides an overview of the product patent application counts, and the extent to which open innovation strategies are adopted by the case firms.

**Table 2:** Patent count and extent of open innovation strategies pursued by pharmaceutical case firms from inception to 2020

Characteristics	Dr. Reddy’s laboratories	Ranbaxy laboratories	Lupin limited	Piramal	Torrent	Advinus	Curadev	Akamara biomedicine
Product Patents filed^i^	More than 75	More than 75	More than 75	51–75	25–50	Less than 25	Less than 25	Less than 25
Research Services	–	±	–	±	–	±	±	±
Crowdsourcing								
Research Partnerships	–	±	–	±	–	–	–	–
Public–Private Partnerships	–	±	±	–	±	–	–	–
In–licensing		–						
Out–licensing	±	–			–		+	
Co–development	+	±	±		–	+	+	

“ + ” indicates that high extent; “–” to a low extent; “ + /–” medium extent. An average of the different types of collaborations was calculated. Values more than the average denote high and less than average denote low. An empty cell indicates a null value

The open innovation agreements used for the analysis are during the period 1995–2021

^i^Count of product patent applications filed between 2005–2020

**Fig. 3:**
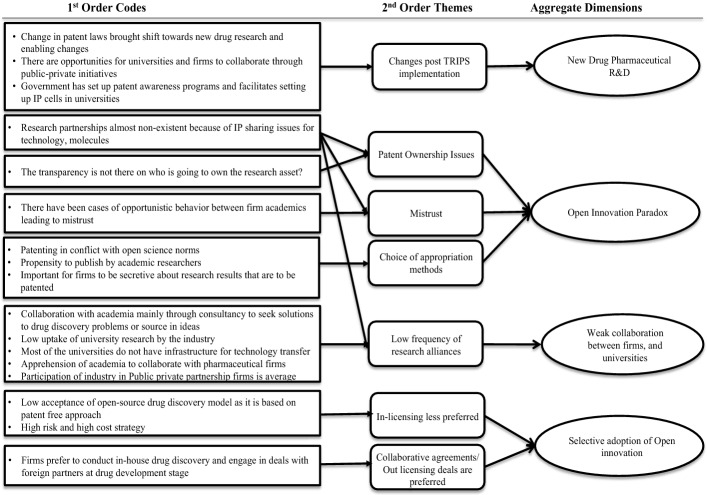
Data Structure

Phase 2—Reconstructing firm level adoption and implementation. Next, we analysed the semi-structured interviews, supplemented with archival data, to understand the adoption of open innovation at firm level. The interviews have been analysed using a variant of thematic analysis within a phenomenological framing, synthesising the descriptive and interpretive traditions (Stierand & Dörfler, 2014). The iterative first-order coding process allowed to capture the meaning units, and to identify patterns in the descriptive findings, aligned with the logic of descriptive phenomenology, showcasing the participants’ viewpoint. Subsequently, these meaning units were synthesized into broader second-order themes and aggregated dimensions, allowing for emergent aspects of interpretive phenomenology, leading to researcher-focused concepts. The process of analysis was visualised using the Gioia framework (Gioia et al., 2013). The first-order codes were discriminated by segregating the interview data into initial list of terms and codes, using in-vivo-terms and phrases used by the participants. In a subsequent round of coding, these terms were further collapsed into higher first-order codes by searching for relationships between the list of codes in order to form meaningful categories. In the second-order analysis, the codes were further collapsed into second-order themes by associating the participants’ accounts with the literature and the research question. During this phase, conceptual links started to emerge between the relevant second-order themes, and these were further combined into fewer and more relevant aggregate dimensions (this is shown on Fig. 3 in the next section).

It is important to note that the insiderness of the lead author also affected the analysis, as the interviewer’s pre-understandings influenced how the codes and themes were formed. These effects are unavoidable and always present to a smaller or greater degree, but we believe that what is important is to acknowledge and examine them in order to keep the findings robust. Therefore, bracketing was practiced throughout the research process through transpersonal reflexivity between the researchers. Bracketing allowed for insiderness to become a source of insight instead of biasing the findings (Dörfler & Stierand, 2021).

We wanted to make sure that our findings are robust. Gummesson (2000) proposes that validity in case study research can be enhanced by integrating the research process with conceptual models which allows the researcher to assess assumptions, constantly revise the results, retest ideas, model and review the limitations of the study. Triangulating sources (e.g. patent data, archival data, interviews) as well as perspectives (interviewees from pharmaceutical firms, universities, and regulatory bodies) allowed for a multidimensional perspective of the phenomenon, enabled corroboration of findings, and enhanced the trustworthiness and reliability of the study (Creswell, 2009; Miles & Huberman, 1994; Patton, 1999). Finally, the findings of the analysis were shared with select interviewees to seek participant validation (Bryman, 2011; Miles & Huberman, 1994); this type of participant validation was particularly useful to enhance the accuracy of the results in case of expert participants.

## Findings

In this section, we present the findings of the study in three stages, following the unfolding of our research topic (cf Stigliani & Ravasi, 2012). First, the insights regarding the open innovation strategies are outlined, to localize the open innovation context in the Indian pharmaceutical sector. Then, we depict what we have learned about the tensions leading to the OIP in the study context. Finally, we present our ideas on integration. The data structure used in the study is shown in Fig. 3.

Throughout this section, we illustrate the narrative with power-quotes from the research participants, which support our emerging insights in Table 7 (Pratt, 2009).

### Strategies for open innovation

India became a signatory to fully implement TRIPS-based laws by January 1, 2005 (Sampat & Shadlen, 2015). Patent statistics show that new patent applications filed by the eight pharmaceutical firms increased from 176 during the period 1995–2004 to 609 during the period 2005–2014, showing an increase of more than 200% (Bhatnagar et al., 2016). The stronger patent laws opened up opportunities for organizations to commercialize their research inventions through various licensing agreements (Arora et al., 2009a). The initiation of research for novel drugs has heralded new opportunities to explore collaborations with public sector researchers for scientific discoveries. The government introduced various incentives, such as increased budgetary allocations for research, soft loans, grants, setting up technology transfer offices, and initiation of PPP initiatives (Department of Science and Technology, 2013; Upadhyay et al., 2010). Public initiatives such as The Drugs and Pharmaceuticals Research programme (DPRP), Biotechnology Industry Research Assistance Council (BIRAC) initiatives and New Millennium Indian Technology Leadership Initiative (NMITLI) support pharmaceutical research in various ways (Joseph, 2011; BIRAC, 2014). The government is also enabling interaction between academics, R&D institutions, and industry by setting up science parks, technology incubation centres, and public private research institutes.

We observed different patterns in the adoption of open innovation from our data analysis. While there is substantial evidence for outbound innovation, only limited use of inbound innovation was observed within the local innovation system. Below, we detail insights regarding the four types of open innovation strategies, namely: research alliance, in-licensing, out-licensing, and co-development used in pharmaceutical research (see Fig. 1).

#### Research alliance

The most prevalent form of research engagements between firms and universities are the research services that involve paid consulting and fee-for-service. We observed that industry scientists consult with academic scientists whenever they require technical help or specific knowledge. The firms may source in research ideas or seek solutions to scientific problems from universities and research institutions. International universities and research institutions are also used sometimes to validate findings or get endorsement for research results (see Tables 2 and 7).

Pharmaceutical professionals in general agree about the benefits of getting academic expertise at the drug discovery phase. When working in a specific disease area, consultation is very useful to understand the nature of the disease and to learn about the research in that area. Interview data reveals that common scientific problems for which consultancy is sought may vary from inactivity of a molecule, failure of a molecule in a cell line or animal model, impurity profiling to stability testing, formulation etc. (see Table 2).

Evidence of non-pecuniary or non-monetary (Dahlander & Gann, 2010) engagements is the interaction with scientists through crowdsourcing platforms. In most cases, membership to these consortiums is free or nominal, and serves as a vital platform to engage with researchers for sourcing knowledge. In India, the CSIR introduced the Open Source Drug Discovery (OSDD) collaborative platform for neglected tropical diseases like tuberculosis, to enable scientists to collaborate through virtual networks for discovery of novel therapies (Årdal & Røttingen, 2012; Bhardwaj et al., 2011). Interestingly, pharmaceutical firms like Lupin are engaged in the research of tuberculosis but are not a part of the OSDD community. This initiative has not managed to get the interest of domestic pharmaceutical firms, which are largely absent from this consortium.

Table 2 shows that Ranbaxy and Piramal are the only two case firms with evidence of long-term R&D collaborations with multiple local entities. Ranbaxy formed research engagements in the initial phases of drug research for discovery of new compounds with various public research institutions such as Anna University, University of Saurashtra, NIPER, and Centre of Biochemical Technology (CBT). Piramal partnered with the Council of Scientific & Industrial Research (CSIR) and the National Institute of Oceanography for the development and screening of natural product library, to identify potential sources of novel drugs. Most of the collaborative projects which have occurred between firms and public institutions are ad hoc and do not extend into long-term research relationships.

Research partnerships with public research institutions through PPP are another route used by pharmaceutical firms to build research networks and get research funds. Ranbaxy and Advinus have formed research alliances with ‘Medicines for Malaria Venture’ (MMV) to develop new treatments for malaria. An initiative by NMITLI involved a successful partnership between 12 public institutions and the pharmaceutical firm Lupin, which led to the development of new tuberculosis drug LL 3858/4858 (Sudoterb). Torrent sought funding assistance through the public initiatives, mostly at drug development phase. PPPs, however, suffer from bureaucracy and are mostly used by pharmaceutical firms to channel research funds.

The general opinion among the research community is that industry tends to focus on in-house drug discovery, despite the presence of various options which firms can use within and outside the local innovation system to source in knowledge (see Table 7). Unlike in the West, where various open innovation models are experimented with by global pharmaceutical firms at exploratory phases of drug discovery (Schuhmacher et al., 2013), the case firms are comparatively closed during the drug discovery phase.

#### In-licensing

The values in Table 2 indicate that in-licensing is not a preferred strategic option among case firms, there are several reasons for this. Firstly, in-licensing of drug compounds is perceived as high risk and costly strategy. Secondly, the lack of experience of purchasing molecules from external sources and the bias of investing funds in drug candidates ‘not-invented’ within the in-house R&D laboratories makes in-licensing a less preferred option. A senior executive of Piramal highlighted the need for due diligence and caution in the case of purchasing molecules, as the risk of failure lies on the purchaser (see Table 7). Thirdly, firms have limited financial resources, which restrains firms’ options to in-license.

“We are open to in-licensing if there are any interesting molecules, but being a start-up company, we cannot pay the millions of dollars that large pharmaceutical can pay.” (Senior Executive, Akamara Biomedicine)

Among the case firms, only Ranbaxy has exercised the in-licensing option and successfully in-licensed a drug called ‘arterolane maleate’ from Medicines for Malaria Venture (MMV), and launched the new drug Synriam in 2012. The first NCE launched in the Indian pharmaceutical landscape through the Ranbaxy-MMV collaboration is a successful exemplar of in-licensing combined with in-house R&D development. The example of Ranbaxy makes a persuasive case to use this open innovation mode in order to leverage the collective competence of a network of external scientists through sourcing or in-licensing route.

#### Out-licensing

The majority of the case firms have adopted out-licensing strategy to generate revenues and commercialise their innovation, as indicated in Table 7. Firms develop molecules in-house in a closed manner and use out-licensing agreements to generate revenue, recover the costs of research and development, and to avoid the risk of late-stage failures. The examples of outbound agreements of the case firms with foreign partners at drug development stage are presented in Table 3.

**Table 3:** Outbound innovation agreements from inception to 2020

Pharmaceutical firm	Phase of Pharmaceutical R&D	Year	Partner firm	Objective of the agreement
Out-licensing				
Torrent	Early Clinical Development	2002	Novartis	Out-licensing of novel drug compound Advanced Glycation End-Products (AGE) Breaker. Option to acquire exclusive global rights for further development and commercialization by Novartis
Ranbaxy	Clinical Development	2002	Shwartz Pharmaceuticals	Ranbaxy out-licensed RBx 258 indicated for the treatment of BPH Exclusive rights to develop, market and distribute the product in US, Japan and Europe to buyer Further development stopped in 2004 by Schwarz Pharma
Ranbaxy	Preclinical phase	2007	Pharmaceutical Product Development (PPD) Inc	Acquisition of exclusive worldwide license by PPD to develop, manufacture and market Ranbaxy's novel statin molecule
Curadev	Drug discovery	2010	US midsized pharmaceutical company	Development by Curadev till drug target identification Transfer of rights to US partner at the candidate selection stage in exchange for milestone payments and royalties
Co-development				
Dr. Reddy’s Laboratories	Preclinical phase	2008	7TM Pharma	Agreement to jointly develop pre-selected targets from the pre-clinical phase up to Clinical Development—Phase IIa
Aurigene (Subsidiary of Dr. Reddy's Laboratories)	Early drug discovery	2015	Curis, Inc	Aurigene to conduct discovery and preclinical activities, IND-enabling studies and Clinical development Phase 1
		2017	Agios Pharmaceuticals	Agreement to research, develop and commercialize small molecule inhibitors of an undisclosed cancer metabolism target
Ranbaxy Laboratories (now Sun Pharmaceutical Industries Ltd.)	Early drug discovery	2003	GSK	Multiyear collaborative deal for research and development of new drugs in the area of respiratory and anti-inflammation
	Preclinical phase	2007	Pharmaceutical Product Development Inc	Acquisition of exclusive worldwide license by PPD to develop, manufacture and market Ranbaxy's novel statin molecule
Lupin Limited	Preclinical phase	2018	AbbVie	Exclusive license agreement to Abbvie for Lupin’s haematological cancer drug, called MALT1. Lupin is eligible to receive milestone-based payments and royalties
	Clinical development	2019	Boehringer Ingelheim	Agreement to develop its MEK inhibitor new drug for difficult-to-treat cancers in exchange for upfront payment, additional payments and entitlement to receive royalties on sales
Torrent Pharmaceuticals	Early drug discovery	2005	AstraZeneca	Research collaboration agreement aimed at discovering a novel drug candidate for hypertension
Advinus	Drug discovery	2006	Merck	Advinus to receive upfront payment and potential milestone payments and royalties for developing clinically validated drug candidates related to metabolic disorders
	Drug discovery	2008	Ortho- McNeil- Janssen Pharmaceuticals	Advinus is responsible for drug discovery and early clinical development until the completion of advanced phase of clinical trials
	Early drug discovery	2014	Takeda Pharmaceutical Company Ltd	Advinus is responsible for leading the programmes to create optimal IND ready compounds for pre-defined targets in the area of inflammation, CNS and metabolic diseases
Curadev Pharma Pvt Ltd	Early drug discovery	2015	Roche	Roche will fund research, development, commercialization costs and provide additional research funding to Curadev’s novel cancer drug
		2019	Takeda Pharmaceutical Company Ltd	Curadev has licensed its novel lead small molecule Stimulator of Interferon Genes (STING) agonist to Takeda
		2020	Bayer Healthcare	The deal is for the use of Curadev’s small molecule Stimulator of Interferon Genes (STING) antagonist programme to identify new drug candidates across lung, cardiovascular and other inflammatory diseases

The examination of the cases over time suggests that the number of out-licensing deals has gone down, and that firms are now moving towards more collaborative agreements for multiple reasons. Firstly, out-licensing limits the licensor’s awards as a proportion potential revenues are transferred out (Reepmeyer, 2006). Secondly, an out-licensing deal at an early phase of drug discovery leads to lower revenues compared to molecules, which are out-licensed at more advanced phases of drug development. Thirdly, out-licensing implies relinquishing control over the molecule. This means that firms can no longer exercise their control if the partner organisation decides to shelve the product at latter phases of drug development. As an example, Torrent out-licensed its novel age-breaker compound to Novartis in 2002, but development for this compound was stopped in 2005. Torrent then re-acquired the rights to the drug and decided to develop in-house. The drug is now in Phase 2 clinical trials for diabetic complications and in phase 3 for heart failure. The risk of losing potential revenues and the inability to retain control influences the choice of the firm. As the Vice-President of a pharmaceutical firm elaborates:

“It depends on the company’s strategy. If you want quick return on investment ..then this is what it takes to have these out-licensing deals. If the molecule succeeds, then you lose the chance to make billions of dollars, but if you fail then by out-licensing at least the company gained something.” (Senior Vice President, Piramal)

#### Co-development

These agreements are an integral part of open innovation strategy for pharmaceutical firms (see Tables 3 and 7). The most common type of collaborative R&D agreement is co-development that allows to share resources and knowledge, and the synergies are expected to lower the risk of the R&D project (Reepmeyer, 2006; Schuhmacher et al., 2016b).

The agreement of Curadev with Roche for their lead cancer compound illustrates the most prominent objectives for pharmaceutical firms to form these agreements: access to funds for R&D in the form of upfront fees and milestone-based payments, mitigating risk with a partner, receiving royalty payments, and gaining territorial commercialization rights. The intellectual property rights in most of these co-development agreements are transferred to the partner firm. The other case firms, Advinus, Aurigene (Dr. Reddy’s), Lupin and Torrent have all entered into multi-year risk-sharing agreements with major firms including Boehringer Ingelheim, Takeda, Roche, Curis, Inc., Merck KGaA etc. for their proprietary in-house drug discovery projects.

“Actually, this is a risk-based business…In terms of collaborative relationship, risk is very less as every aspect is mutually agreed and foreseen further. But in terms of progress of the project, that risk is always there; as you know drug discovery is very risky.”(Senior Vice President, Piramal)

Interview data indicates that typically, pharmaceutical firms undertake internal proprietary drug discovery research up to the pre-clinical phase, and then seek a co-development alliance with a foreign multinational firm for clinical development and commercialisation. The main advantage of co-development is that the partners monitor the progress of drug molecule mutually and take joint decisions. Co-development arrangements have become the most popular way of advancing the cost-intensive risk-prone drug discovery programs among the local pharmaceutical firms.

### Tension in open innovation

The previous section shows that the case firms engage predominantly in outbound innovation to commercialise their innovation, however their efforts vary regarding inbound innovation. The analysis of the case firms’ collaborative agreements reveals that the extent of R&D collaborations between academic research and industry is low among Indian pharmaceutical firms (see Table 2). We found three barriers to the formation of open innovation networks: a) choice of appropriability methods b) patent ownership and c) mistrust, which is discussed in detail below.

#### Choice of appropriability methods

Patents are the most common form of appropriability mechanism used by pharmaceutical firms. However, researchers in the universities and public research labs have the propensity to publish their research work avoiding the patent route. The decision of academics to publish instead of patent is driven by an open science attitude, philanthropic motives, and by fulfilling established norms for promotion. Academic inventors often believe that public research is funded by taxpayers’ money and they in turn are obliged to ensure that the fruits of the research flow back into society. Many academics feel that patenting conflicts with the open science norms associated with the rapid disclosure of research results and an environment of knowledge sharing (see Table 7).

This difference in opinion between academics and industry causes much friction. In industry, scientists recognize the need to be secretive about research results, which are to be patented, and take precautions to prevent information leakage; otherwise, it becomes prior art, and the invention will have no value (Senior Executive, Ranbaxy).

“For scientists in public research labs, publications are more important for their progress. Companies are not interested in publication; patents and research output are more important for our progress.” (Senior Executive, Piramal)

Most of the universities in India do not have a formal infrastructure in place to allow academic researchers to avail opportunities for commercial utilisation of scientific research. The most prominent institutions in India, such as IIT Delhi and NIPER, have established a technology transfer department, which enables patenting and facilitates knowledge transfer activity from the university to firms. However, in many universities, the concept of patenting is still new.

The government has initiated patent awareness programs and IP departments are being set up across the nation. The National Research Development Council (NRDC, 2016) also facilitates patenting to university researchers for a small fee. The Biotechnology Industry Research Assistance Council (BIRAC), which has many public–private initiatives, feels the need for strengthening the patent infrastructure in universities in order to remove impediments to collaborative efforts. Increasingly, universities are now realizing the need for patenting, and steps are being taken to catch up in this direction.

#### Patent ownership

The sharing of intellectual property rights poses a bigger problem for cultivating collaborative relationships. The general finding is that firms do not want to share patents with academic institutions. They are willing to collaborate with academics as consultants, but they are hesitant to engage in long-term projects that might result in patent sharing agreements. As the director of a public partnership initiative points out, the trend by pharmaceutical firms is to in-license a technology or knowledge from academics, and to undertake in-house research in silos (see Table 7). This allows firms to retain control of their research work (Advisor, Ministry of Science and Technology). The feedback from industry professionals and experts underlines the importance placed by firms on retaining control of research assets within the firm for potential out-licensing deals.

“The issue is the ownership of the technology, molecules, the ownership of any kind of platform they are developing. The transparency is not there on who is going to own? If the assets are coming from a pharmaceutical company, they feel that academic is just doing a service. An academic professor says that he is not doing just a service. He is helping you to understand what a molecule does in the biology field.” (Senior Executive, Pfizer)

An industry expert notes that research partnerships are almost non-existent because of IP sharing issues (Expert, Drug Discovery Research).

#### Mistrust

The decades of disconnect during the process patent regime between the industry and academia has distanced the innovating entities. Research collaborations between these two sectors in the past have been tainted with sporadic events of opportunism and disagreements over patent ownership, which have led to bias and mistrust between the two sectors. This has substantial effect on future negotiations and undermines the primary purpose of collaborative relationship (see Table 7). Discussions with academics and firms have pinpointed many cases of opportunistic behaviour where the academics felt cheated as they have not been given their due share.

“Our own nanoxel, which is now commercial, actually came from a university. Of course that was also ridden with certain controversies […] Again mistrust. So, it did come from collaboration, but it wasn’t the best of collaborations and the IP was completely ours. (Vice President, Dabur Research Foundation)

Firms engage with the academic community through personal networks, which allow them to build trust through personal interactions and leverage the research expertise. Such interactions serve as control mechanism to reduce conflicts related to patent sharing and ownership. The director of a PPP initiative asserts that one of the positive effects of patent regime has been the availability of opportunities for universities and firms to collaborate through public–private initiatives. However, as most of this early-stage research is related to intellectual property development work, it is difficult to tackle the apprehension the academia has in collaborating with the pharmaceutical firms (Advisor, BIRAC). These observations indicate the problems related to disclosure and theft of research ideas have negatively swayed the attitudes of universities and public research institutions when interacting with the industrial sector.

These findings highlight the specific challenges firms in developing countries face in balancing the tension between intellectual protection and openness. The need to protect proprietary knowledge, specifically in the early phases of research, negatively impacts openness and collaboration between the entities. An important deciding factor for firms is whether to open the pharmaceutical R&D to external partners at early phases of research to speed up innovation, or to remain closed to ensure intellectual property rights and increase a firm’s ability to capture profit from innovation. This gives rise to the open innovation paradox.

### Integration opportunities

A comparative assessment of open innovation strategies between pharmaceutical case firms with global leading firms is provided in Table 4. The comparative findings show that global pharmaceutical firms use inbound open innovation extensively during drug discovery for sourcing in knowledge, and employ various outbound arrangements to commercially exploit their innovation in drug development phases. Meanwhile, case firms use inbound open innovation selectively in the drug discovery phase, and enter into licensing and co-development agreements with external partners only during drug development.

**Table 4:** Comparison of open innovation strategies of case pharmaceutical firms with global pharmaceutical firms

Open innovation strategies	Pharmaceutical case firms		Global pharmaceutical firms	
	Extent of open innovation	Examples	Extent of open innovation	Examples
Research Services	High engagements with academics mostly through fee for service projects through personal networks	Not Available	Allows to generate new ideas and solve specific discovery related problems	Not Available
Crowdsourcing	Negligible use of crowdsourcing platforms	Open Source Drug Discovery (OSDD)	High level of uptake through crowdsourcing platforms	Grants4Targets by Bayer – Partners: AstraZeneca, Eli Lilly, GSK Innocentive and Open Innovation Drug Discovery by Eli Lily – Partners: AstraZeneca, GSK Open innovation platform—Astra Zeneca The Synaptic Leap's Schistosomiasis (TSLS)- GSK
Research Partnerships	Evidence of low frequency of long term research partnerships Beneficial for knowledge sourcing In-licensing opportunity	CSIR—Piramal Drug Validation Project Medicines for Malaria Venture (MMV)—Partners include Ranbaxy (2003) and Advinus (2008)	Used extensively for screening and lead optimization of their molecules, discovery of new drug compounds	Phenotypic Drug Discovery (PD2) and Target based screening (TargetD2) by Eli Lily
Public–Private Partnerships	Uptake for PPP is average however not the core business for many firms Beneficial to Speed up innovation Research funding Collaborative research	New Millennium Indian Technology Leadership Initiative (NMITLI) – Lupin	Used by global pharmaceutical firms to pool resources for research in neglected disease Social benefits Patent pools used to grant licenses to partners	Medicines for Malaria Venture (MMV)—Partners include GSK, Novartis, Sanofi, Janssen, Merck KGaA and Takeda The Global Alliance for Tuberculosis Drug Development (TB Alliance)—Partners include GSK, Bayer, Novartis Drugs for Neglected Diseases Initiative (DNDi)—Partners include Astra Zeneca, Bayer AG, Boehringer Ingelheim, Novartis, Sanofi, GSK, Johnson & Johnson, Pfizer, Roche
In-licensing	Not used extensively due to: Expensive and risk option Limited financial resources Lack of experience Not- invented -here syndrome	In-licensing of a drug ‘arterolane maleate’ by Ranbaxy from Medicines for Malaria Venture (MMV)	Preferred mode to in-license new drugs/technology/patents Also suffers from managerial bias of not-invented here	Pfizer's Pregabalin (Lyrica) originated in Northwestern University Pfizer’s palbociclib (Ibrance) had its origins at Warner-Lambert and Onyx Pharmaceuticals J&J’s Infliximab (Remicade) was synthesized at New York University J&J’s Abiraterone (Zytiga) originated at UK Institute of Cancer Research
Out-licensing	Frequently used Allows commercialization of innovation	Out-licensing deal of Curadev with US company for milestone payments and royalties	Preferred option for drug candidates with low estimated returns Enables to manage investments in multiple projects	Merck out-licenses Phase IIb-ready atacicept to Vera Therapeutics Merck KGaA has an out-licensing agreement with Novartis for osteoarthritis clinical-stage programme
Co-development	Most frequently used Allows to share risks/costs/resources and profits	Curadev deal with Bayer (2020) Advinus- Takeda Pharmaceutical deal Boehringer Ingelheim deal with Lupin	Frequently used to gain competitive advantage and expand portfolio Allows to share risks/costs/resources and profits	Boehringer Ingelheim and Eli Lilly have co-development deals for: Jardiance (empagliflozin) Trajenta (linagliptin) Basaglar (insulin glargine) AstraZeneca and Merck to co-develop Lynparza (olaparib) for multiple cancer types Novartis has formed co development deals for: Cardiovascular with Ionis Pharma Eye indications with Tribos

Specialised knowledge resulting from university research constitutes an important source of innovation, however the dominance of traditional academic norms and associated secrecy required in collaborative R&D adversely influence scientists' motivation to collaborate with private firms (Perkmann & West, 2014; Perkmann et al., 2013). In addition, pharmaceutical research dominated by high levels of patenting stimulates secrecy among the firms, and adversely affects external collaborations leading to OIP (Arora et al., 2016; Laursen & Salter, 2005, 2014). In developing countries, these challenges are more prominent coupled with poor infrastructure of tech transfer systems, lack of policies such as the Bayh Dohl Act, and low patenting propensity among academics. This is paradoxical for firms who want to protect their intellectual property to commercialize later through licensing agreements and seek exclusive ownership of IP.

The examination of open innovation strategies of global pharmaceutical firms has deepened our understanding on how global best practices can be adopted in pharmaceutical research to overcome OIP. The intellectual protection strategies of firms to protect the value of research can have a cascading effect on the adoption of innovation strategy, inhibiting the kind of external collaborations important for pharmaceutical research. The analysis of global pharmaceutical firms shows how they effectively employ various practices such as continuous exploration of research opportunities with a wide network of scientists, selective revealing, committed research partnerships with universities, and setting up a diligent process to continuously evaluate drug candidates developed outside the organization. The interviews with experts and archival data of case firms show that Indian pharmaceutical firms have employed many of these strategies rather sporadically. This assessment allowed us to derive key requirements for effective integration opportunities for developing countries, namely to help overcome tensions related to protection of intellectual property research. Ideally, this is done in a way that is context independent, such that it can be applied across various knowledge domains and in various developing countries that face similar challenges. Table 5 shows how pharmaceutical firms in developing countries can amalgamate open innovation approaches with in-house R&D, and offers insights to navigate the OIP.

**Table 5:** Framework for integration of open innovation in pharmaceutical R&D in developing countries

Open innovation strategies	Challenges (Paradox)	Mode of collaboration	Integration opportunities for developing countries
Research alliance	Fear of knowledge spillovers Limited research scope of contractual projects Limited research networks to personal contacts	Research Services Crowdsourcing platforms	Expand network of scientists and researchers Continuously explore opportunities to expand open innovation networks Selective revealing to solve specific drug discovery problems to prevent knowledge spillovers
	Academic collaborations results in exclusive patent rights to the innovator or shared patent rights Requires academic scientists to delay knowledge spillovers and secrecy till patents are filed	Research partnerships Public–Private Partnerships Research Consortiums	Invest in research partnerships with well-defined contractual agreements Establish partnerships to leverage expertise and explore in-licensing opportunities Participation in drug collaborative projects would enable to gain confidence for reproducibility of results and reduce not-invented-here syndrome Capitalize on research opportunities for neglected diseases
In-licensing	Expensive option Lack of experience	In-licensing from local/external universities, Startups/Biotechnology firms Research partnerships	Set up a dedicated process to screen and evaluate drug candidates Invest time and effort to screen and evaluate drug candidates This can enable to speed up innovation and fill up the product pipeline
Out-licensing	The need to patent promotes secrecy and restricts openness Loss of ownership rights	Collaborative agreement with a foreign partner	Out-license to manage costs and risks Consider outsourcing in late phases of development for increased revenue potential Consider outsourcing if risk for the company is high and estimated returns are low Ensure profit from innovation
Co-development	The need to patent promotes secrecy and restricts openness Engagement with an external partner may lead to loss of control and market exclusivity	Collaborative agreement with a foreign partner	Co-develop with external partner to share costs, risks and profits Employ this strategy for drug candidates with high return potential Late phase agreements would ensure high returns Ensures profiting, risk sharing and control

Bringing together our empirical research and the extant literature, we identify five practices that pharmaceutical firms in developing countries can adopt to leverage the benefits of open innovation; these are discussed below.

#### Expand the network of scientists and researchers

Using various approaches such as crowdsourcing platforms, conferences, virtual consortiums. It has been well established in literature that academic collaborations can facilitate sourcing in ideas, get expert opinions, help solve complex drug discovery problems, and facilitate organisational learning through knowledge exchange between in-house scientists and external researchers. By using selective revealing techniques (Foege et al., 2019), firms can avoid the risk of unintended knowledge spillovers whilst safeguarding proprietary knowledge.

#### Invest in research partnerships with well-defined contractual agreements

that define clear exclusivity, patent ownership, and information disclosure clauses (Perkmann & West, 2014). Leading global pharmaceutical firms enter into research agreements with universities and research consortiums to explore attractive drug candidates for in-licensing and to ensure a pipeline of innovation drug compounds. PPPs also facilitate patent pooling and sharing of resources to accelerate innovation in diseases that mainly afflict developing countries.

#### Set up a dedicated process to screen and evaluate drug candidates

Developed by external partners to select potential candidates. This can speed up innovation and fill up the product pipeline. Lilly’s Phenotypic Drug Discovery Initiative uses this innovation strategy to explore various in-licensing opportunities. Merck has in-licensed 17% of its portfolio pipeline while GSK, Pfizer, Roche, and Novartis have in-licensed more than 8% of their R&D pipeline from external sources over a period of 18 years from 1996 to 2013. Most of the high potential drug candidates are in-licensed from universities (O'Connell et al., 2014).

#### Out-license to manage costs and risks

Firms should assess the commercial value of the compound and use this strategy to effectively manage competing projects. Industry experts recommend that outsourcing should be considered in late phases of development to yield high revenues for firms.

#### Co-develop with external partners to share costs, risks and profits

Firms in developing countries should evaluate the variables of risk, phase of drug development, research costs, and potential value of the drug candidate to leverage profitable sharing agreements. Pharmaceutical industry experts opine that firms can use these agreements to bargain for control in decision making, access complementary assets such as distribution channels, and gain co-promotion rights in certain territories (Bianchi et al., 2011; Schuhmacher et al., 2016b).

## Discussion

Prior research has pointed out the existence of a paradox of openness (Arora et al., 2016; Laursen & Salter, 2005, 2014; Ritala & Stefan, 2021), which is particularly significant when innovation is technologically complex and relies heavily on patenting (Almirall & Casadesus-Masanell, 2010; Wang et al., 2017). In this paper, we addressed OIP in the adoption of open innovation in the pharmaceutical R&D for new drugs in the context of a developing country. Our study of the eight Indian pharmaceutical firms in the pharmaceutical sector explored how a firm’s open innovation strategy varies by stages of pharmaceutical research.

Pharmaceutical firms have traditionally relied on in-house R&D with minimal interaction with universities and research labs to support their new development process. Our analysis documents important changes in their approach: (1) firms in our sample have gradually adopted open innovation approaches in different phases of drug research and moved away from a closed innovation model, (2) outbound innovation through licensing agreements is the most preferred strategy that generates revenues from IP assets, and (3) firms face tension in opening up to external partners without intellectual protection in drug discovery phase. The tension that firms face in employing open innovation modes are driven by the need to control ownership of research assets, ensure secrecy, and avoid conflicts related to intellectual protection and patent ownership. Pharmaceutical case firms employ open innovation selectively by restricting inbound innovation when protection of research assets is not guaranteed, and use outbound innovation during drug development phases to leverage their intellectual protection. This approach allows them to balance the tensions between patenting and openness. This is in line with the previous findings by Laursen and Salter (2014) and Granstrand and Holgersson (2017) who suggest that in order to avoid the conflicts over control and ownership of knowledge in external collaborations, firms practice selective revealing; partially disclosing relevant knowledge while maintaining secrecy (Foege et al., 2019).

Global pharmaceutical firms have established new models of open innovation that allow navigating the open innovation paradox and managing the dimensions of patenting and openness across all phases of R&D. Firms in a developing country face additional challenges such as open science norms in universities, mistrust, and patent ownership issues, which makes it even more challenging to adopt open innovation. Though traditional academic norms and values still more or less dominate or influence scientists’ motivation to collaborate with private firms even in developed countries Perkmann et al., (2013), after the implementation of the Bayh Dohl Act, university systems are shifting to proprietary science through patenting, and commercialization of research output with industry partners. However, in developing countries, academic scientists emphasise openness and transparency and prefer to publish their research findings to get promotions, secure grants, and propel additional research in line with the open science norms. Weak local open innovation networks and the continuation of open science norms in academics cause firms to be wary of academic collaborations. In order to reduce opportunistic behaviour, pharmaceutical firms leverage external expertise through personal networks and mainly through contract-based fee-for-service projects, which limit the project scope and knowledge sharing. Technology transfer systems are now functional in leading Indian universities such as IIT, NIPER, which have set up technology transfer cells, alongside significant budgetary allocation by the governments to stimulate collaborative R&D. These science-based investments are important to propel development and sharing of new ideas and to encourage opportunity-based entrepreneurship (Fini & Sobrero, 2020) for commercialising scientific knowledge.

The selective use of open innovation practices among the case firms shows that there is a compelling need to strategically harmonise control and openness in intellectual protection to leverage benefits of open innovation and co-creation (Tekic & Willoughby, 2019). The data from global pharmaceutical firms shows how firms employ open innovation at early phase of drug discovery research to build open innovation networks, ensure a robust pipeline of new drugs, and increase R&D efficiency while managing appropriability issues.

Ultimately, by combining extant theoretical understanding with our empirical findings, we conceptualize an integrative framework to expand the scope of open innovation in pharmaceutical R&D and accelerate the adoption of open innovation strategies in developing countries’ pharmaceutical sector. Firms in developing countries can adopt the following practices to integrate open innovation with in-house R&D: (1) Expand their networks of scientists and researchers (2) Set up a dedicated process to screen and evaluate drug candidates (3) Invest in research partnerships with well-defined contractual agreements (4) Out-license to manage costs and risks, and (5) Co-develop with external partner to share costs, risks and profits. These insights can support firms to navigate the tensions of patenting and openness in pharmaceutical R&D process.

In recent times, the response to the COVID 19 pandemic brought together multiple innovation systems and has opened up a myriad of possibilities for firms and policymakers for commercialization of science with a broader societal impact of their research through open innovation (Chesbrough, 2020; Fini et al., 2018). The emergency response to the pandemic has forced the pharmaceutical sector to explore various open innovation initiatives, such as patent pooling, sharing metadata, genetic pooling and repository sharing among scientists across the world. The integrated global clinical trials and the partnering of regulatory authorities to accelerate the regulatory approval process for COVID-19 vaccines is a testimony how firms can leverage open innovation and yet profit from innovation (Ahn et al., 2019; Enkel et al., 2009). The efforts of multinational firms like Eli Lily, Merck, GlaxoSmithKline and Pfizer provide useful insights for developing countries to draw together knowledge from different contributors to develop and exploit innovation.

## Conclusion

This paper contributes to scholarly literature on open innovation, particularly the challenges of balancing openness and protection of intellectual property. The study focuses on pharmaceutical R&D in a developing country, India. Empirical findings enable us to understand at which stages of the new drug R&D process firms pursue closed innovation, and what factors encourage a more open approach. The paper compares the practices of Indian pharmaceutical companies with the open innovation models of top global pharmaceutical firms that use these strategies to gain advantage in innovation without compromising on intellectual protection or profitability aspects. This assessment allowed the researchers to analyze the advantages and disadvantages of different forms of open innovation strategies, and helps to understand the paradoxical challenges in the adoption of open innovation in pharmaceutical R&D.

By synthesizing lessons from existing open innovation models adopted by global firms, this article introduces a framework for pharmaceutical firms in developing countries to mitigate the tensions arising from OIP and allows profiting from innovation. In this way, this study can enable senior management of pharmaceutical firms to navigate OIP in practice and adopt the suggested open innovation practices to build new drug pipelines, and propel research in a cost-effective manner. The specific findings highlighted in this research provide a holistic view of the problems inherent in the sector important for policy makers to formulate bespoke interventions to promote public private interactions between science and industry.

As far as the limitations of the paper are concerned, the findings are obtained from the innovation practices of a pharmaceutical industry that faces its unique set for drivers and constraints. As such, the framework extended in this study is not tested for usability in other sectors or industries. As we have demonstrated, our empirical findings build upon the earlier literature in this field which shows that openness and patenting are conflicting dimensions that require careful consideration (Laursen & Salter, 2014). Specifically, in a developing country setting there are additional factors in play that affect boundary decisions such as mindset, history of weak collaborations between university and industry, prevalence of open science norms, and poor technology transfer mechanisms. However, it is possible that there may be other causes at play, or there may be deeper underlying causes other than the highlighted issues which may accumulate and hinder collaboration. A further investigation in this line of research may help to get an enhanced understanding of the science industry interactions and is another promising direction for future research.

Looking forward and acknowledging the value of using specialized knowledge from external sources for pharmaceutical innovation, further insights are needed to accelerate knowledge sharing. Future research in the area of communities of practices (CoP)—defined as communities where members invest their identities in order to learn together and from each other about problems that they genuinely care about (Brown & Duguid, 1991; McDermott, 2000; Nicolini et al., 2022; Pyrko et al., 2017, 2019; Wenger, 1998)—can be an important area for coping with the open innovation paradox, particularly in areas where academic-practitioner collaboration is useful, such as in the case of OSDD community. We find evidence in our research that managerial biases towards openness, and adoption of ideas ‘not-invented’ within the organization can be important influencing factors for a firm’s open innovation strategy. The issue of how managerial attitudes enable and constrain open innovation appears to be an interesting area for future research. Increased policy emphasis by the Indian government to implement various public initiatives and set up technology transfer offices in universities could boost the trend towards increased university patenting. Future research efforts in this direction can provide broader insights on how academic entrepreneurship (Shibayama et al., 2012) can shape academic norms and outcomes (Siegel et al., 2003) for innovation collaborations.

The experience and strategies of the Indian pharmaceutical firms provides important lessons for developing countries that have stepped up efforts in new drug innovation. A possible future research avenue could be to understand the specific challenges in other developing countries and explore the relationship of culture in influencing the shift from standalone innovation to a more network based socially efficient innovation framework. We believe that this paper represents a valuable basis for future research as well as managerial discussions in the field.

## Appendix: list of interview participants

**Table:**

Position	Name of organization
Professor	University of Mysore
Professor	University of Hyderabad
Professor	University of Pune
Professor	Dr. Bhanuben Nanavati College of Pharmacy
Professor	Saurashtra University
Professor and Head	National Institute of Pharmaceutical Education and Research—NIPER
Professor	National Institute of Pharmaceutical Education and Research—NIPER
Professor	Jamia Hamdard
Professor	IIT Delhi
Associate Director	Foundation for Innovation and Technology Transfer at IIT Delhi
Dean	IIT Delhi
Professor	Jawaharlal Nehru University
Professor	All India Institute of Medical Sciences—AIIMS
Scientist	National Chemical Laboratory
Scientist	Indian Institute of Chemical Technology
Scientist	Central Drug Research Institute
Professor	Dr. Reddy's Institute of Life Sciences—DRILS
Sr Scientist	National Chemical Laboratory
Project Director	Open Source Drug Discovery—OSDD
Advisor	Department of Science and Technology – DST
Advisor	Biotechnology Industry Research Assistance Council—BIRAC
Scientist	Open Source Drug Discovery—OSDD
Head	New Millennium Indian Technology Leadership Initiative—NMITLI
Assistant Director	The Federation of Indian Chambers of Commerce and Industry -FICCI
Manager	National Research Development Corporation—NRDC
Chief Scientific Officer	Curadev Pharma Private Limited
Ex Vice President Formulations	Ranbaxy Laboratories Limited
Ex-President	Dr. Reddy's Laboratories
Senior Vice President	Lupin Limited
Senior Group Leader	Piramal life sciences
Senior Vice President	Piramal life sciences
Senior Vice President	Piramal life sciences
Vice President	Piramal life sciences
General Manager	Torrent pharmaceuticals limited
Chief Scientific Officer	Advinus
Vice President	Lupin Limited
Associate Director	Dr. Reddy's laboratories
Senior Vice President	Ranbaxy laboratories limited
Associate Director	Daiichi Sankyo
Director	Akamara Biomedicine
Managing Director	Lifecare Innovations
Assistant Director	Ara Healthcare (Biopharmaceutical firm)
Director	Novo Informatics (Spinoff IIT Delhi)
Vice President	Dabur Research Foundation (Contract Research Organization)
Vice President	Fresenius Kabi Oncology Limited (Generics company)
Associate Director	Jubilant Chemsys (Contract Research Organization)
General Manager	Alkem (Generics company with NCE division)
Senior Director	Pfizer
Sr Director, Research	Center for Advanced Drug Research, SRI International
Chief Scientific Officer	Thinq Pharma, India

See Tables 6 and 7.

**Table 6:** Overview of data sources and usage in the analysis

Type of data	Data source	Use in the analysis
Patent data	Patent database of India, US and WIPO 780 patent applications were analysed to estimate product patent application count	Product patent application count as an indicator of firm’s innovation output
Archival Data	Firm related documents: Annual reports, firm websites, press releases in online magazines – Pharmabiz, Express Pharma Public initiatives documents and project list of four programs – DPRP, BIRAC, OSDD and NMITLI Data from available literature on open innovation strategies of top 20 global pharmaceutical firms identified by market capitalization	Tracking of research alliances formed by case firms with universities and public research institutes to understand local innovation networks Tracked and compiled firm history for events, research partnerships, agreements etc Compiled a list of open innovation agreements of global pharmaceutical firms categorised by strategy type and stage of pharmaceutical research Used to understand the extent of open innovation in Table 2 and list types of outbound agreements used in Table 3 Support, integrate and triangulate evidence from interviews
Interviews	50 interviews were conducted during the period 2014–2016 The breakdown is as follows: •17 interviews with senior management of firms •19 interviews with academic researchers and scientists •5 with public department officials •9 interviews with experienced professionals in Indian pharmaceutical sector	Familiarize with the open innovation context in pharmaceutical sector Integrate with evidence of collaborative networks to gain understanding of open innovation strategies of firms with local and foreign entities Understand the challenges of open innovation

The top 20 global pharmaceutical firms have been selected based on their market capitalization as of Jan 1, 2020 (https://www.value.today/world-top-companies/pharmaceutical).

**Table 7:** Selected Quotes that supported our emergent interpretation

2nd order themes	Selected quotes on 1st order codes
Changes post TRIPS implementation	Change in patent laws brought shift towards new drug research and positive changes “In terms of financial incentives, we have got new grants from the government to help us develop novel chemical entities” (Chief Scientific Officer, Advinus)
	There are opportunities for universities and firms to collaborate through public–private initiatives “Scientists who are proactive in their research don’t know what to do after that and where to go to seek help. BIRAC has come forward now to help them to a great extent” (Advisor, BIRAC)
	Government has set up patent awareness programs and facilitates setting up IP cells in universities “We really have to set up these technology transfer officers and IP officers across the country to help the university researchers” (Advisor, BIRAC)
Patent Ownership Issues	Research partnerships almost non-existent because of IP sharing issues for technology, molecules “In my view, there is more fluff than wheat. The projects are not substantial like in Europe and United States. The collaborating partners make an agreement, have a press release, they would say how much money was invested and that is well and good. But the thing is what has come out of these collaborations?” (Senior Director, SRI International)
	Transparency on ownership of research assets “There is a serious mind-set problem in collaborating. When two or three people are collaborating in a project; even before an idea has generated and work has been initiated, they begin to worry about and squabble on how they will divide their gains and achievements” (Professor, University of Hyderabad)
	There is low acceptance of open-source drug discovery model as it is based on patent free approach “OSDD is almost IP neutral. Our main contention is that IP with a monopolistic or exclusivity led approach is of no value in diseases without market…. what we are trying to do is to develop an innovation model which will work in a situation where markets fail to work.” (Project Director, OSDD)
Mistrust	There have been cases of opportunistic behavior between firm academics leading to mistrust “The apprehension among academics is that if we work with an industry somebody will take away our IP and we would not have any control on it.” (Advisor, BIRAC)
	Firms and academics do not prefer to collaborate with each other “When I came to India, I saw that the academic sector and private sector do not see eye to eye. This is very strange to me as abroad this is very common. Somehow those two sectors are moving in their own manner, do not meet each other.” (Professor, DRILS)
Choice of appropriation methods	Patenting in conflict with open science norms “I am not crazy about patents. Patenting is for revenue and recognition. If my clients are happy with my work, that itself is a reward for me.” (Professor, University of Mysore)
	Propensity to publish by academic researchers “Universities are not geared up on intellectual property (IP) part […] as far as IP infrastructure in the university is concerned it is still evolving not developed at this moment.” (Retd. Vice President Formulations, Ranbaxy)
	Important for firms to be secretive about research that are to be patented “It is in the nature of the business. I am not in the business of trusting. I am in the business of protecting my assets […]. Intellectual property is all about secrecy and I have to protect that secrecy, it’s not open source.” (Chief Scientific Officer, Curadev)
Low frequency of research alliances	Collaboration with academia mainly through consultancy to seek solutions to drug discovery problems or source in ideas “We had few molecules and lots of patents but there are no takers because nobody is interested in new molecules. We have various collaborations with industry for contractual work or consultancy work but no joint collaborative work for new molecule research is going on.” (Professor, NIPER) “We sought help from IIT Kanpur from a professor in organic chemistry. But the collaboration was for a very focused and specific problem.” (Chief Scientific Officer, Curadev)
	Low uptake of university research by the industry “Very often our findings are left at the work bench …the institution can take the lead further in directions required to reach the end user. But that is not happening.” (Professor, Jawaharlal Nehru University)
	Most of the universities do not have infrastructure for technology transfer “Universities are not geared up on intellectual property (IP) part […] as far as IP infrastructure in the university is concerned it is still evolving not developed at this moment.” (Retd. Vice President Formulations, Ranbaxy)
	Apprehension of academia to collaborate with pharmaceutical firms “When I came to India, I saw that the academic sector and private sector do not see eye to eye. This is very strange to me as abroad this is very common. Somehow those two sectors are moving in their own manner, do not meet each other.” (Professor, DRILS)
	Limited participation of industry in Public private partnership firms My personal opinion is that industry seems less responsive as they feel that if it is a profit making successful programme then why not do it ourselves. The industry does not have shortage of funds so they think why should we have an academic partner..” (Professor and Head of Department, NIPER)
In-licensing less preferred	High risk and high cost strategy “In case of in-licensing, you are buying somebody’s molecule and putting in your own money to develop that to completion. So, whenever you are purchasing or in-licensing any molecule… due diligence activities have to be done very carefully before you could in license these projects.” (Senior Group Leader, Piramal)
Collaborative/ Out- licensing agreements are preferred	Firms prefer to conduct in-house drug discovery and engage in deals with foreign partners at drug development stage “We have a pipeline of molecules that we are working on out-licensing and hopefully that will happen soon.” (Chief Scientific Officer, Advinus) “Most of the agreements are co-development where companies undertake research together. In the end, there will be various clauses like, if it comes to phase 1, the partner will give X million and if it goes to phase 2, it will be Y million. There will be also agreement that if the drug comes to the market, the Indian company may have rights to market in India or Asia.” (Professor, University of Hyderabad)

## Footnotes

1 Appropriability is an innovator’s ability to capture return on its invention (Arrow, 1962).

2 World Health Organization defines a generic drug as “a pharmaceutical product, usually intended to be interchangeable with an innovator product, that is manufactured without a license from the innovator company and marketed after the expiry date of the patent or other exclusive rights” (Alfonso-Cristancho et al., 2015).

3 The notion that a technology or research asset cannot be relied upon for its quality and performance if not produced inside a company is referred to as NIH (Chesbrough, 2003a, b).

4 Government sponsored initiatives include The Drugs and Pharmaceuticals Research programme (DPRP), New Millennium Indian Technology Leadership Initiative (NMITLI), Biotechnology Industry Research Assistance Council – BIRAC and Open Source Drug Discovery—OSDD.

5 The top 20 global pharmaceutical firms have been selected based on their market capitalization as of Jan 1, 2020 (https://www.value.today/world-top-companies/pharmaceutical).
